# 1-Allyl-2-amino­pyridin-1-ium bromide

**DOI:** 10.1107/S1600536813012452

**Published:** 2013-05-15

**Authors:** T. Seethalakshmi, P. Venkatesan, M. Nallu, Daniel E. Lynch, S. Thamotharan

**Affiliations:** a Department of Physics, Government Arts College (Autonomous), Karur 639 005, India; b School of Chemistry, Bharathidasan University, Tiruchirappalli 620 024, India; c Faculty of Health and Life Sciences, Coventry University, Coventry CV1 5FB, England; d Department of Bioinformatics, School of Chemical and Biotechnology, SASTRA University, Thanjavur 613 401, India

## Abstract

In the cation of the title salt, C_8_H_11_N_2_^+^·Br^−^, the dihedral angle between the planes of the pyridinium ring and the allyl group is 79.4 (3)°. In the crystal, N—H⋯Br and weak C—H⋯Br hydrogen bonds link the cations and anions, forming chains of alternating *R*_2_^1^(7) and *R*_4_^2^(8) rings, which run parallel to the *c*-axis direction. The crystal studied was an inversion twin with components in a 0.753 (12):0.247 (12) ratio.

## Related literature

For related structures, see: Seethalakshmi *et al.* (2006*a* ▶,*b* ▶,*c* ▶, 2007 ▶, 2013 ▶). For the biolgical activity of alkyl-pyridinium salts, see: Sundararaman *et al.* (2013 ▶); Ilangovan *et al.* (2012 ▶). For hydrogen-bond graph-set motifs, see: Bernstein *et al.* (1995 ▶).
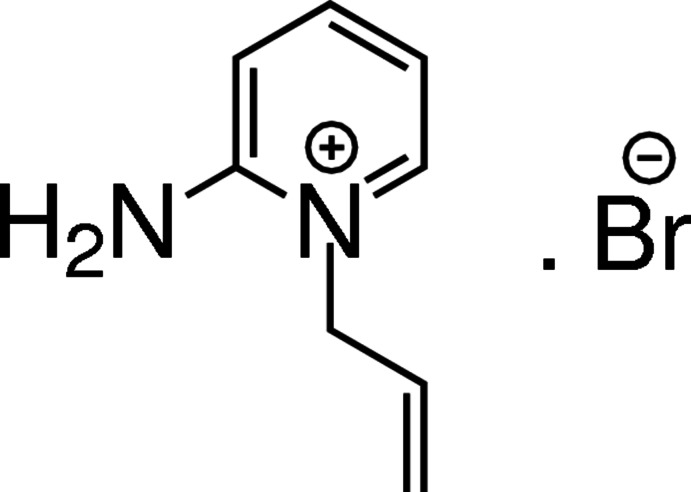


## Experimental

### 

#### Crystal data

C_8_H_11_N_2_^+^·Br^−^
*M* *_r_* = 215.10
Orthorhombic, 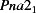

*a* = 7.8205 (2) Å
*b* = 13.3560 (3) Å
*c* = 8.5621 (2) Å
*V* = 894.32 (4) Å^3^
*Z* = 4
Mo *K*α radiation
μ = 4.53 mm^−1^
*T* = 120 K
0.30 × 0.14 × 0.03 mm

#### Data collection

Bruker–Nonius 95mm CCD camera on κ-goniostat diffractometer
Absorption correction: multi-scan (*SADABS*; Sheldrick, 2003 ▶) *T*_min_ = 0.343, *T*_max_ = 0.876
14540 measured reflections
2033 independent reflections
1960 reflections with *I* > 2σ(*I*)
*R*_int_ = 0.058

#### Refinement

*R*[*F* ^2^ > 2σ(*F* ^2^)] = 0.023
*wR*(*F* ^2^) = 0.053
*S* = 1.06
2033 reflections
110 parameters
3 restraints
H atoms treated by a mixture of independent and constrained refinement
Δρ_max_ = 0.43 e Å^−3^
Δρ_min_ = −0.35 e Å^−3^
Absolute structure: Flack (1983 ▶), 945 Friedel pairs
Flack parameter: 0.247 (12)

### 

Data collection: *COLLECT* (Nonius, 1998 ▶); cell refinement: *DENZO* (Otwinowski & Minor, 1997 ▶); data reduction: *DENZO*; program(s) used to solve structure: *SIR92* (Altomare *et al.*, 1994 ▶); program(s) used to refine structure: *SHELXL97* (Sheldrick, 2008 ▶); molecular graphics: *PLATON* (Spek, 2009 ▶); software used to prepare material for publication: *SHELXL97*.

## Supplementary Material

Crystal structure: contains datablock(s) I, global. DOI: 10.1107/S1600536813012452/lh5611sup1.cif

Structure factors: contains datablock(s) I. DOI: 10.1107/S1600536813012452/lh5611Isup2.hkl

Supplementary material file. DOI: 10.1107/S1600536813012452/lh5611Isup3.cml

Additional supplementary materials:  crystallographic information; 3D view; checkCIF report

## Figures and Tables

**Table 1:** Hydrogen-bond geometry (Å, °)

*D*—H⋯*A*	*D*—H	H⋯*A*	*D*⋯*A*	*D*—H⋯*A*
N2—H2*A*⋯Br1^i^	0.84 (2)	2.61 (2)	3.412 (2)	160 (4)
N2—H2*B*⋯Br1^ii^	0.85 (2)	2.51 (2)	3.357 (2)	175 (3)
C6—H6*A*⋯Br1^iii^	0.99	2.91	3.668 (3)	134
C6—H6*B*⋯Br1^i^	0.99	2.84	3.810 (3)	167

Symmetry codes: (i) 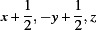; (ii) 
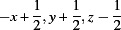
; (iii) 
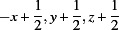
.

**Fig. 1.**
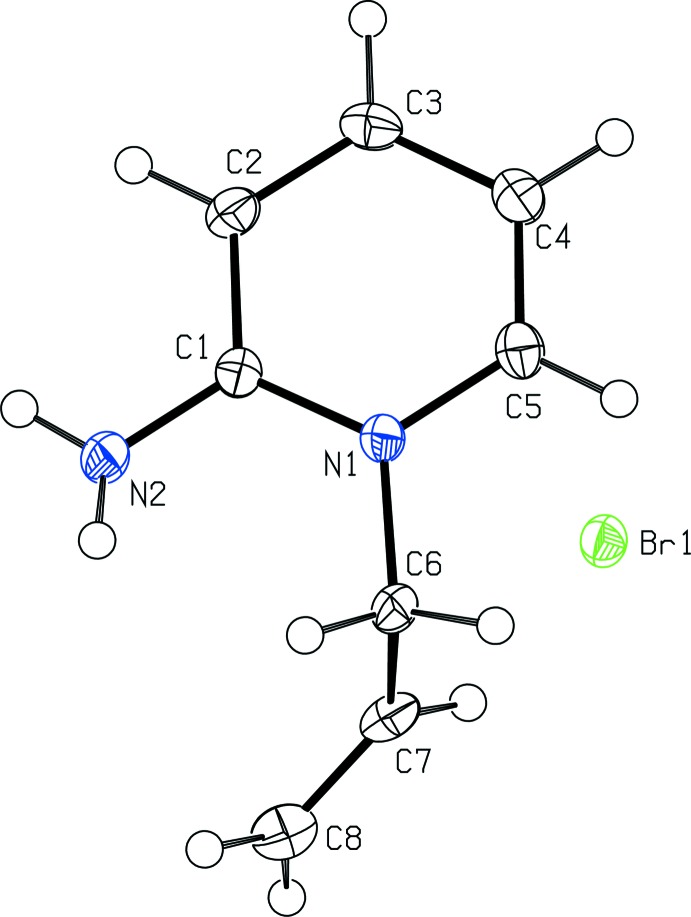
Molecular structure of (I), showing ellipsoids at the 50% probability level.

**Fig. 2.**
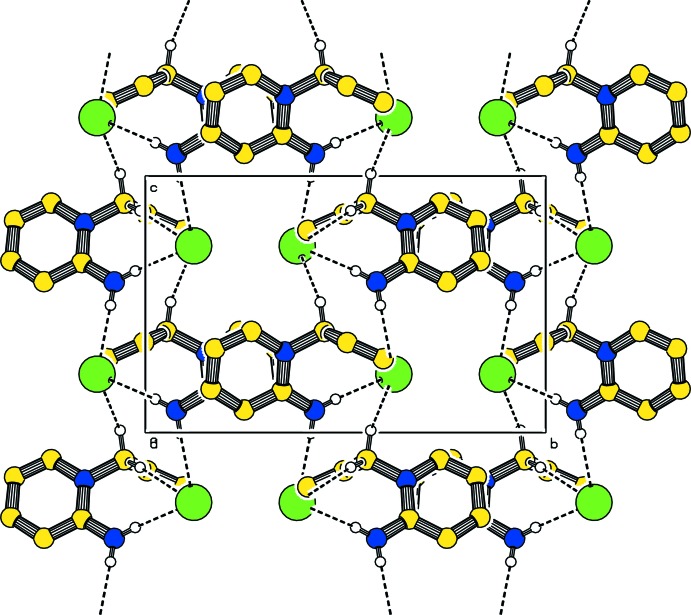
Part of the crystal structure of (I) viewed along the a axis. The hydrogen bonds are indicated as dashed lines.

**Fig. 3.**
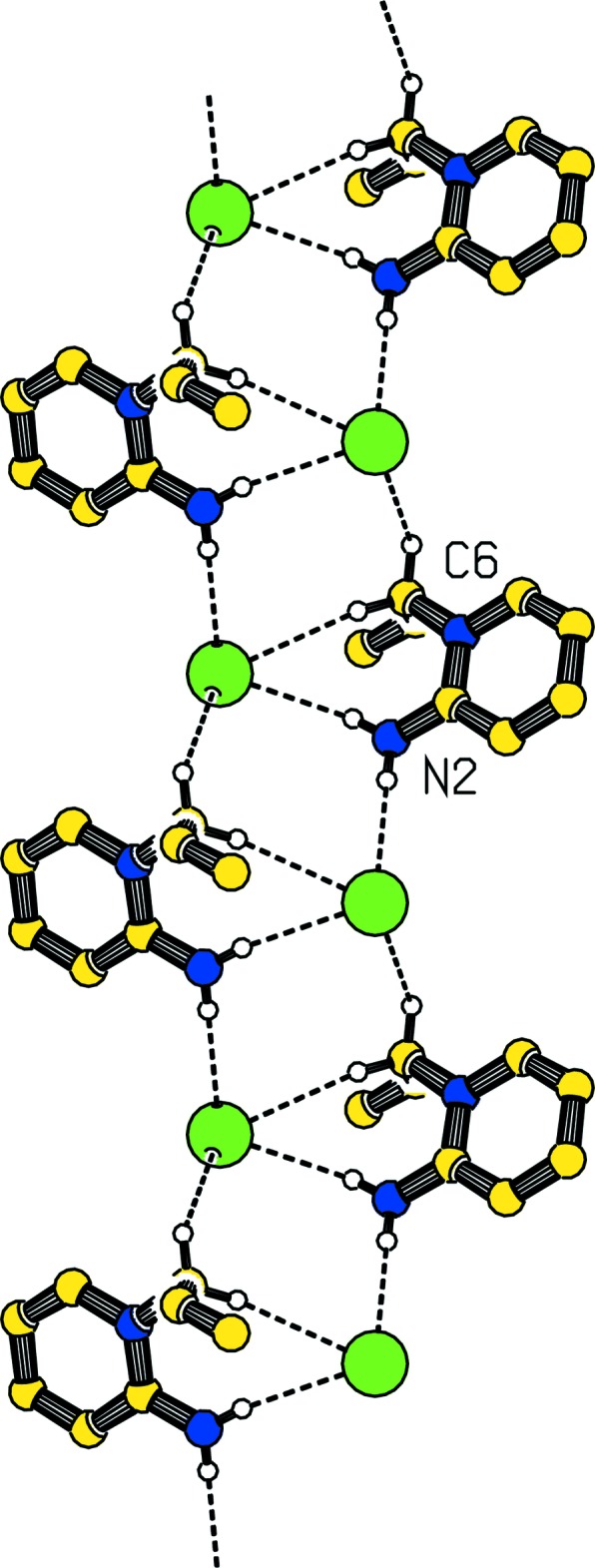
Arrangement of alternate R12(7) and R24(10) ring motifs in a one-dimensional chain.

## supplementary crystallographic information

### Comment

As part of our studies on pyridinum salts (Seethalakshmi *et al.*, 2006*a*,*b*,*c*, 2007, 2013), we report herein the crystal structure of the title compound, (I). The asymmetric unit of (I) is shown in Fig. 1. The dihedral angle between the planes of the pyridinium ring and allyl group (C6/C7/C8) is 79.4 (3)°. The corresponding bond lengths and angles of the cation in (I) are comparable with those of related structures reported earlier (Seethalakshmi *et al.*, 2006*a*,*b*,*c*, 2007, 2013).

In the crystal (Fig. 2) the amino group acts as a donor for two different bromide anions (Table 1). These intermolecular N—H···Br hydrogen bonds link the cations *via* bromide anions into one-dimensional chains which run parallel to the *c* axis. In addition, weak intermolecular C—H···Br interactions are observed between C6 (*via* H6A and H6B) and two bromide anions. The N2—H2A···Br1^i^ and C6—H6B···Br1^i^ interactions combine to generate a *R*^1^_2_(7) ring (Bernstein *et al.*, 1995) and two N—H···O hydrogen bonds and two C—H···Br interactions combine to form a *R*^2^_4_(8) ring motif. These two ring motifs are arranged alternately and run parallel to the *c* axis (Fig. 3).

### Experimental

A solution of 2-aminopyridine (1.175 g, 25 ml) and allyl bromide (1.51 g, 25 ml) in dry acetone (15 ml) was stirred for 44 h at room temperature (303 K). The solid that separated was filtered, washed with dry acetone and dried in vacuum to give the stable salt, which was recrystallized from an aqueous ethanol (80% *v*/*v*) solution (m.p. 419–421 K, yield 63%).

### Refinement

The positions of amino H atoms were determined from a difference Fourier map and refined freely along with their isotropic displacement parameters. In the final round of refinement, the N—H bond lengths of amino group were restrained to 0.86 (2) Å. The remaining H atoms were placed in geometrically idealized positions (C—H = 0.95–0.99 Å), with *U*_iso_(H) = 1.2*U*_eq_(C) and were constrained to ride on their parent atoms. The crystal used is an inversion twin with components in the ratio 0.753 (12):0.247 (12)

### Crystal data

**Table:**

C_8_H_11_N_2_^+^·Br^−^	*F*(000) = 432
*M**_r_* = 215.10	*D*_x_ = 1.598 Mg m^−^^3^
Orthorhombic, *P**n**a*2_1_	Mo *K*α radiation, λ = 0.71073 Å
Hall symbol: P 2c -2n	Cell parameters from 1211 reflections
*a* = 7.8205 (2) Å	θ = 2.9–27.5°
*b* = 13.3560 (3) Å	µ = 4.53 mm^−^^1^
*c* = 8.5621 (2) Å	*T* = 120 K
*V* = 894.32 (4) Å^3^	Plate, colourless
*Z* = 4	0.30 × 0.14 × 0.03 mm

### Data collection

**Table:**

Bruker–Nonius 95mm CCD camera on κ-goniostat diffractometer	2033 independent reflections
Radiation source: Bruker-Nonius FR591 rotating anode	1960 reflections with *I* > 2σ(*I*)
Graphite monochromator	*R*_int_ = 0.058
Detector resolution: 9.091 pixels mm^-1^	θ_max_ = 27.5°, θ_min_ = 3.0°
φ and ω scans	*h* = −10→10
Absorption correction: multi-scan (*SADABS*; Sheldrick, 2003)	*k* = −17→17
*T*_min_ = 0.343, *T*_max_ = 0.876	*l* = −10→11
14540 measured reflections	

### Refinement

**Table:**

Refinement on *F*^2^	Hydrogen site location: inferred from neighbouring sites
Least-squares matrix: full	H atoms treated by a mixture of independent and constrained refinement
*R*[*F*^2^ > 2σ(*F*^2^)] = 0.023	*w* = 1/[σ^2^(*F*_o_^2^) + (0.0198*P*)^2^ + 0.704*P*] where *P* = (*F*_o_^2^ + 2*F*_c_^2^)/3
*wR*(*F*^2^) = 0.053	(Δ/σ)_max_ = 0.001
*S* = 1.06	Δρ_max_ = 0.43 e Å^−^^3^
2033 reflections	Δρ_min_ = −0.35 e Å^−^^3^
110 parameters	Extinction correction: *SHELXL97* (Sheldrick, 2008), Fc^*^=kFc[1+0.001xFc^2^λ^3^/sin(2θ)]^-1/4^
3 restraints	Extinction coefficient: 0.0051 (7)
Primary atom site location: structure-invariant direct methods	Absolute structure: Flack (1983), 945 Friedel pairs
Secondary atom site location: difference Fourier map	Flack parameter: 0.247 (12)

### Special details

**Table:**

Experimental. The minimum and maximum absorption values stated above are those calculated in *SHELXL97* from the given crystal dimensions. The ratio of minimum to maximum apparent transmission was determined experimentally as 0.611792.
Geometry. All e.s.d.'s (except the e.s.d. in the dihedral angle between two l.s. planes) are estimated using the full covariance matrix. The cell e.s.d.'s are taken into account individually in the estimation of e.s.d.'s in distances, angles and torsion angles; correlations between e.s.d.'s in cell parameters are only used when they are defined by crystal symmetry. An approximate (isotropic) treatment of cell e.s.d.'s is used for estimating e.s.d.'s involving l.s. planes.
Refinement. Refinement of *F*^2^ against ALL reflections. The weighted *R*-factor *wR* and goodness of fit *S* are based on *F*^2^, conventional *R*-factors *R* are based on *F*, with *F* set to zero for negative *F*^2^. The threshold expression of *F*^2^ > σ(*F*^2^) is used only for calculating *R*-factors(gt) *etc*. and is not relevant to the choice of reflections for refinement. *R*-factors based on *F*^2^ are statistically about twice as large as those based on *F*, and *R*- factors based on ALL data will be even larger.

### Fractional atomic coordinates and isotropic or equivalent isotropic displacement parameters (Å^2^)

**Table:**

	*x*	*y*	*z*	*U*_iso_*/*U*_eq_	
Br1	0.03564 (3)	0.120651 (14)	0.72785 (7)	0.01951 (9)	
C1	0.2522 (4)	0.6591 (2)	0.6626 (3)	0.0171 (6)	
C2	0.2481 (4)	0.75344 (18)	0.5884 (3)	0.0223 (5)	
H2	0.2826	0.7598	0.4825	0.027*	
C3	0.1942 (4)	0.8354 (2)	0.6696 (4)	0.0238 (6)	
H3	0.1909	0.8989	0.6198	0.029*	
C4	0.1434 (4)	0.8267 (2)	0.8268 (4)	0.0227 (6)	
H4	0.1071	0.8837	0.8841	0.027*	
C5	0.1474 (4)	0.7353 (2)	0.8941 (3)	0.0210 (5)	
H5	0.1121	0.7285	0.9997	0.025*	
C6	0.1943 (3)	0.55284 (19)	0.8940 (3)	0.0195 (5)	
H6A	0.1970	0.5632	1.0085	0.023*	
H6B	0.2962	0.5131	0.8647	0.023*	
C7	0.0363 (3)	0.4961 (2)	0.8511 (3)	0.0221 (6)	
H7	−0.0714	0.5267	0.8704	0.026*	
C8	0.0379 (4)	0.4061 (2)	0.7883 (4)	0.0264 (6)	
H8A	0.1437	0.3737	0.7678	0.032*	
H8B	−0.0666	0.3736	0.7635	0.032*	
N1	0.2010 (3)	0.65157 (19)	0.8142 (3)	0.0174 (5)	
N2	0.3083 (3)	0.57779 (17)	0.5869 (2)	0.0208 (5)	
H2A	0.339 (5)	0.524 (2)	0.630 (4)	0.049 (11)*	
H2B	0.341 (5)	0.588 (3)	0.494 (3)	0.040 (10)*	

### Atomic displacement parameters (Å^2^)

**Table:**

	*U*^11^	*U*^22^	*U*^33^	*U*^12^	*U*^13^	*U*^23^
Br1	0.02238 (13)	0.02017 (12)	0.01599 (12)	−0.00267 (8)	−0.00157 (17)	0.00066 (16)
C1	0.0158 (12)	0.0185 (14)	0.0168 (12)	−0.0021 (11)	−0.0001 (10)	−0.0016 (10)
C2	0.0245 (13)	0.0228 (14)	0.0196 (13)	−0.0015 (11)	0.0013 (11)	0.0045 (10)
C3	0.0259 (15)	0.0163 (13)	0.0293 (15)	0.0019 (12)	−0.0013 (11)	0.0036 (10)
C4	0.0223 (14)	0.0220 (14)	0.0238 (15)	0.0013 (11)	0.0000 (11)	−0.0053 (11)
C5	0.0225 (14)	0.0230 (14)	0.0176 (12)	0.0010 (11)	−0.0009 (11)	−0.0037 (10)
C6	0.0255 (13)	0.0198 (12)	0.0131 (11)	0.0014 (10)	0.0020 (10)	0.0024 (9)
C7	0.0196 (13)	0.0238 (14)	0.0228 (14)	−0.0012 (10)	0.0014 (10)	0.0097 (11)
C8	0.0240 (14)	0.0286 (14)	0.0265 (13)	−0.0040 (11)	−0.0018 (10)	0.0051 (12)
N1	0.0211 (12)	0.0169 (11)	0.0141 (11)	−0.0002 (10)	0.0004 (10)	−0.0004 (9)
N2	0.0265 (12)	0.0215 (12)	0.0144 (11)	0.0023 (9)	0.0006 (9)	0.0014 (9)

### Geometric parameters (Å, º)

**Table:**

C1—N2	1.339 (4)	C6—N1	1.486 (3)
C1—N1	1.361 (3)	C6—C7	1.496 (4)
C1—C2	1.412 (4)	C6—H6A	0.9900
C2—C3	1.364 (4)	C6—H6B	0.9900
C2—H2	0.9500	C7—C8	1.317 (4)
C3—C4	1.408 (4)	C7—H7	0.9500
C3—H3	0.9500	C8—H8A	0.9500
C4—C5	1.350 (4)	C8—H8B	0.9500
C4—H4	0.9500	N2—H2A	0.844 (18)
C5—N1	1.376 (4)	N2—H2B	0.849 (19)
C5—H5	0.9500		
			
N2—C1—N1	119.9 (3)	C7—C6—H6A	109.3
N2—C1—C2	120.9 (3)	N1—C6—H6B	109.3
N1—C1—C2	119.2 (3)	C7—C6—H6B	109.3
C3—C2—C1	119.6 (3)	H6A—C6—H6B	108.0
C3—C2—H2	120.2	C8—C7—C6	123.7 (3)
C1—C2—H2	120.2	C8—C7—H7	118.2
C2—C3—C4	120.5 (3)	C6—C7—H7	118.2
C2—C3—H3	119.7	C7—C8—H8A	120.0
C4—C3—H3	119.7	C7—C8—H8B	120.0
C5—C4—C3	118.4 (3)	H8A—C8—H8B	120.0
C5—C4—H4	120.8	C1—N1—C5	120.2 (3)
C3—C4—H4	120.8	C1—N1—C6	120.9 (3)
C4—C5—N1	122.0 (3)	C5—N1—C6	118.8 (2)
C4—C5—H5	119.0	C1—N2—H2A	125 (3)
N1—C5—H5	119.0	C1—N2—H2B	115 (3)
N1—C6—C7	111.5 (2)	H2A—N2—H2B	118 (4)
N1—C6—H6A	109.3		
			
N2—C1—C2—C3	−178.4 (3)	C2—C1—N1—C5	−0.5 (4)
N1—C1—C2—C3	0.4 (4)	N2—C1—N1—C6	−4.5 (4)
C1—C2—C3—C4	0.2 (4)	C2—C1—N1—C6	176.6 (3)
C2—C3—C4—C5	−0.8 (4)	C4—C5—N1—C1	0.0 (4)
C3—C4—C5—N1	0.7 (4)	C4—C5—N1—C6	−177.2 (3)
N1—C6—C7—C8	122.7 (3)	C7—C6—N1—C1	−79.8 (3)
N2—C1—N1—C5	178.3 (3)	C7—C6—N1—C5	97.4 (3)

### Hydrogen-bond geometry (Å, º)

**Table:**

*D*—H···*A*	*D*—H	H···*A*	*D*···*A*	*D*—H···*A*
N2—H2*A*···Br1^i^	0.84 (2)	2.61 (2)	3.412 (2)	160 (4)
N2—H2*B*···Br1^ii^	0.85 (2)	2.51 (2)	3.357 (2)	175 (3)
C6—H6*A*···Br1^iii^	0.99	2.91	3.668 (3)	134
C6—H6*B*···Br1^i^	0.99	2.84	3.810 (3)	167

Symmetry codes: (i) *x*+1/2, −*y*+1/2, *z*; (ii) −*x*+1/2, *y*+1/2, *z*−1/2; (iii) −*x*+1/2, *y*+1/2, *z*+1/2.
